# Semiautomated segmentation of lower extremity MRI reveals distinctive subcutaneous adipose tissue in lipedema: a pilot study

**DOI:** 10.1117/1.JMI.10.3.036001

**Published:** 2023-05-15

**Authors:** Shannon L. Taylor, Paula M. C. Donahue, Michael D. Pridmore, Maria E. Garza, Niral J. Patel, Chelsea A. Custer, Yu Luo, Aaron W. Aday, Joshua A. Beckman, Manus J. Donahue, Rachelle L. Crescenzi

**Affiliations:** a Vanderbilt University, Department of Biomedical Engineering, Nashville, Tennessee, United States; b Vanderbilt University Medical Center, Department of Physical Medicine and Rehabilitation, Nashville, Tennessee, United States; c Vanderbilt University Medical Center, Dayani Center for Health and Wellness, Nashville, Tennessee, United States; d Vanderbilt University Medical Center, Department of Radiology and Radiological Sciences, Nashville, Tennessee, United States; e Vanderbilt University Medical Center, Department of Neurology, Nashville, Tennessee, United States; f Vanderbilt University Medical Center, Department of Pediatrics, Nashville, Tennessee, United States; g Vanderbilt University Medical Center, Vanderbilt Translational and Clinical Cardiovascular Research Center, Division of Cardiovascular Medicine, Nashville, Tennessee, United States; h Vanderbilt University Medical Center, Department of Psychiatry, Nashville, Tennessee, United States

**Keywords:** lipedema, body composition, musculoskeletal segmentation, whole-body magnetic resonance imaging, chemical-shift-encoded magnetic resonance imaging

## Abstract

**Purpose:**

Lipedema is a painful subcutaneous adipose tissue (SAT) disease involving disproportionate SAT accumulation in the lower extremities that is frequently misdiagnosed as obesity. We developed a semiautomatic segmentation pipeline to quantify the unique lower-extremity SAT quantity in lipedema from multislice chemical-shift-encoded (CSE) magnetic resonance imaging (MRI).

**Approach:**

Patients with lipedema (n=15) and controls (n=13) matched for age and body mass index (BMI) underwent CSE-MRI acquired from the thighs to ankles. Images were segmented to partition SAT and skeletal muscle with a semiautomated algorithm incorporating classical image processing techniques (thresholding, active contours, Boolean operations, and morphological operations). The Dice similarity coefficient (DSC) was computed for SAT and muscle automated versus ground truth segmentations in the calf and thigh. SAT and muscle volumes and the SAT-to-muscle volume ratio were calculated across slices for decades containing 10% of total slices per participant. The effect size was calculated, and Mann–Whitney U test applied to compare metrics in each decade between groups (significance: two-sided P<0.05).

**Results:**

Mean DSC for SAT segmentations was 0.96 in the calf and 0.98 in the thigh, and for muscle was 0.97 in the calf and 0.97 in the thigh. In all decades, mean SAT volume was significantly elevated in participants with versus without lipedema (P<0.01), whereas muscle volume did not differ. Mean SAT-to-muscle volume ratio was significantly elevated (P<0.001) in all decades, where the greatest effect size for distinguishing lipedema was in the seventh decade approximately midthigh (r=0.76).

**Conclusions:**

The semiautomated segmentation of lower-extremity SAT and muscle from CSE-MRI could enable fast multislice analysis of SAT deposition throughout the legs relevant to distinguishing patients with lipedema from females with similar BMI but without SAT disease.

## 1 Introduction

**Fig. 1:**
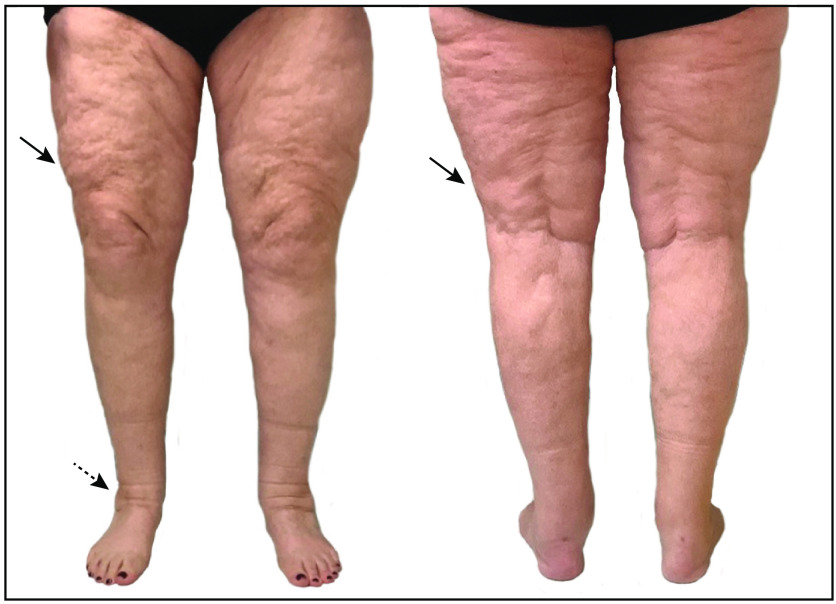
A female with a typical lipedema external presentation. Both anterior and posterior views show the appearance of columnar legs. Other features include skin dimpling (solid arrows) and ankle cuffing (dashed arrow). Though lipedema may have notable external features, patients can be misdiagnosed or mismanaged as obesity, motivating development of objective methods to differentiate lipedema.

Lipedema is a chronic disease marked by abnormal adipose tissue that almost exclusively impacts women.^1^ Lipedema typically presents with excessive subcutaneous adipose tissue (SAT) deposition, symmetrical lower extremity enlargement that spares the feet, subcutaneous nodular tissue texture, and pain in the affected areas.^2^^–^^5^ Lower extremity SAT deposition is often disproportionate causing a columnar appearance in the legs with dimpling of the skin (Fig. 1). Lipedema is often misdiagnosed as obesity and is generally unrecognized as a distinct clinical condition. Unlike nonlipedema fat accumulation, lipedema-related fat accumulation is refractory to dietary, exercise, pharmacologic, and surgical weight loss interventions.^3^^–^^6^ Patients can have both nonlipedema- and lipedema-related fat accumulation, which adds to the complexity of diagnosis. The ability to make a clinical diagnosis of lipedema is further complicated by the lack of quantitative measures to characterize and differentiate lipedema, and this remains a critical unmet need in the field.^5^^,^^7^

Magnetic resonance imaging (MRI) is a noninvasive, clinically feasible modality with contrast between adipose and nonadipose tissues in the legs. Chemical-shift-encoded (CSE) MRI uses a multiecho sequence to image fat- and water-bound proton species simultaneously, and their distinct precession rates are exploited to generate separate fat-weighted and water-weighted images.^8^ CSE contrast is suitable for accurate, reproducible quantification of adipose tissue and skeletal muscle.^9^^–^^13^ Prior CSE-MRI studies showed a distinct amount of SAT deposition in the calves of patients with lipedema compared to women without lipedema who had similar body-mass-index (BMI), although SAT deposition is known to occur throughout the legs in lipedema.^14^^,^^15^ CSE-MRI can be acquired in a whole-body manner to potentially aid quantification of SAT distribution in lipedema, as well as reveal which regions of the legs provide optimal distinguishing biomarkers from obesity.

In this paper, we aimed to develop and validate a novel semiautomated image analysis pipeline for lower extremity CSE-MRI acquisitions and subsequently test the hypothesis that SAT volume is uniquely elevated and distributed throughout the legs of participants with lipedema compared to control participants without lipedema matched for BMI.

## 2 Methods

### 2.1 Participants

All participants (n=28) provided informed consent in accordance with the Vanderbilt University Medical Center Institutional Review Board. We aimed to evaluate our methods over a wide range of BMIs, and as such participants were required to have a BMI within the range of 18.0 to $40.0\;\mathrm{kg}/m^{2}$. Enrolled participants with lipedema (n=15) were required to meet the following primary inclusion criteria as evaluated by a Lymphedema Association of North America-certified physical therapist (experience = 19 years): bilateral and symmetric enlargement of the legs, negative Stemmer’s sign, and clinical criteria for stage and type of lipedema (adopted from Herbst^5^). At least one of the following secondary criteria of lipedema was also required: lower extremity pain, family history of lipedema, nonpitting lower extremity edema, easy bruising, or hypermobility. Control participants (n=13) were enrolled with similar age, BMI, sex, and race as lipedema participant characteristics. Exclusion criteria for all participants were current skin infections, signs of acute inflammation, history of uncontrolled hypertension, diabetes, arthritis, or MRI contraindications.

### 2.2 MRI Acquisition

Participants underwent an MRI exam in the head-first supine position on a 3.0T scanner (Philips Ingenia R5.3, Philips Healthcare; Best, The Netherlands). Whole-body CSE-MRI, also called the Dixon method,^8^ was performed in eight stacks from head-to-ankles, using a 3D, dual-echo spoiled gradient echo sequence that was acquired with the body coil for proton radiofrequency transmission and reception. For each stack, 84 slices were acquired with the following parameters: repetition time = 3.83 ms, echo time 1 = 1.15 ms, echo time 2 = 2.30 ms, field-of-view $(\mathrm{FOV})=530\times 347.8\times 252\;{\mathrm{mm}}^{3}$, $\text{spatial resolution}=2.07\times 1.35\times 3.0\;{\mathrm{mm}}^{3}$, water-fat shift = 0.353 pixels. Parameters were adapted from the AMRA Medical Body Composition Profile protocol (AMRA Medical, Linköping, Sweden).^16^ Cumulative scan duration was ∼6  min. Each CSE-MRI acquisition and reconstruction produced four image contrasts: in-phase, out-of-phase, water-weighted, and fat-weighted.

### 2.3 Image Segmentation

Image analysis focused on the lower extremity stacks from the whole-body MRI acquisitions to quantify tissue composition metrics in regions primarily affected by lipedema. The upper- and lower-transverse slice limits of the image volume were manually selected using standard anatomical landmarks in the superior thigh (slice below the ischial tuberosity at the lesser trochanter of the femur neck) and inferior ankle (lowermost slice containing Achilles’ tendon above widening of the tibia). Duplicate slices resulting from overlap of neighboring stacks were removed.

**Fig. 2:**
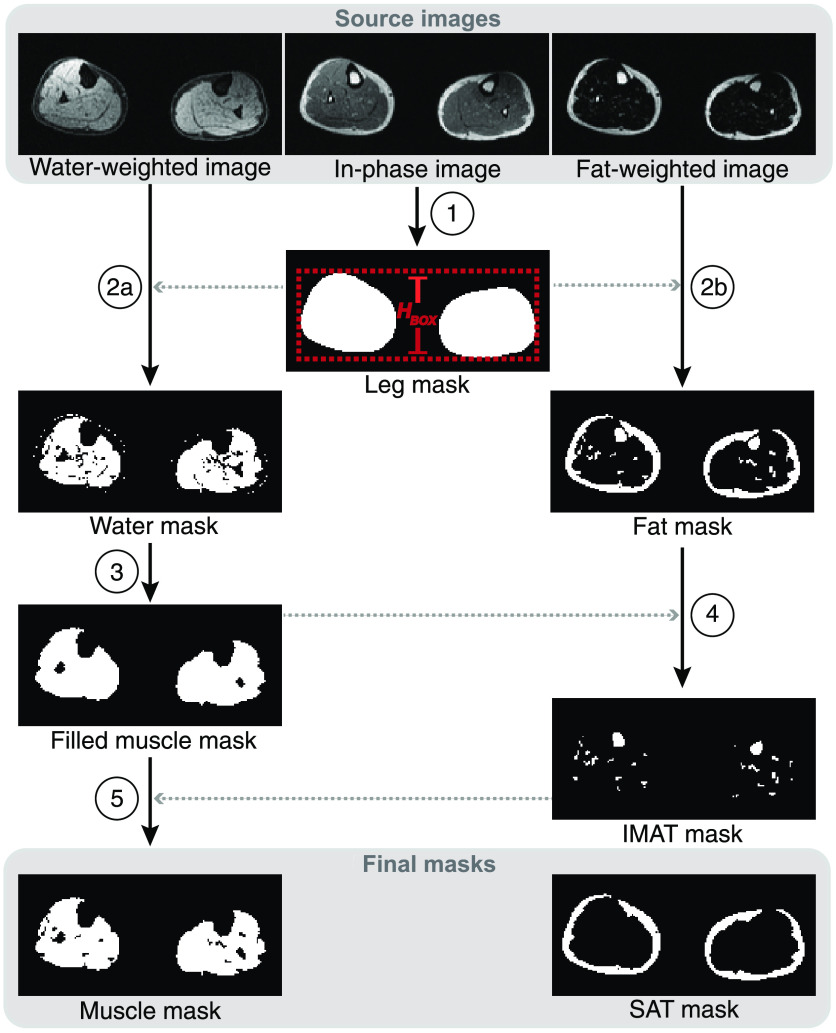
Schematic of automated segmentation. A mask of the leg boundaries is created using active contours segmentation of the in-phase image in step (1). Transverse slices of the water-weighted and fat-weighted images are masked by the leg boundary prior to classification of water and fat pixels in step (2a) and (2b), respectively. Classification is performed with adaptive thresholding with a custom-defined kernel size for each slice, based on the bounding box (red box) height ($H_{\mathrm{BOX}}$) in step (1). Morphological operations and flood-filling are applied to the water mask to label the skeletal muscle region in step (3) and produce a filled muscle mask. The fat mask is compartmentalized into marrow and IMAT and the SAT with a series of Boolean and morphological operations in step (4), to produce a final SAT mask. The marrow/IMAT structures are removed from the filled muscle mask in step (5) to produce a final muscle mask. Gray dotted arrows indicate steps where masks obtained from the other contrast were used. The top gray box delineates the source image contrasts (water-weighted, in-phase, and fat-weighted) used in segmentation, and the bottom gray box shows final masks (muscle and SAT) used for subsequent volume quantification.

Fat- and water-weighted images were segmented to partition the SAT and muscle regions of interest (ROIs) while excluding bone marrow and intermuscular adipose tissue (IMAT), in a multislice routine using the MATLAB Image Processing Toolbox (R2022b, MathWorks; Natick, Massachusetts, United States) (Fig. 2). Methods were applied on each 2D transverse slice in the lower extremity image volume. The automated segmentation algorithm can be considered as five steps: (1) leg boundary definition, (2) fat- or water-dominant voxel classification, (3) muscle identification, (4) adipose tissue compartmentalization, and (5) IMAT and bone marrow extraction.

#### 2.3.1 Leg border definition

For each axial slice in the image volume, a binary mask bound by the leg perimeter was created by performing active contour Chan-Vese segmentation [activecontour(), iterations = 100, MATLAB R2022a] on the in-phase image contrast^17^ (Fig. 2, step 1). Active contours segmentation was chosen for its iterative framework that curves around an object’s boundary, in this case suitable for identifying the edge boundary of the two legs in the image foreground. The in-phase images provided high contrast between the legs and background, with less contrast between internal structures that might confound the active contouring, and this contrast performed better than the fat- and water-weighted images in leg boundary detection. The leg mask was applied to both the fat-weighted and water-weighted images to exclude other anatomy appearing in the FOV (i.e., arms or hands) before the following processing steps.

#### 2.3.2 Adaptive thresholding for fat/water classification

**Fig. 3:**
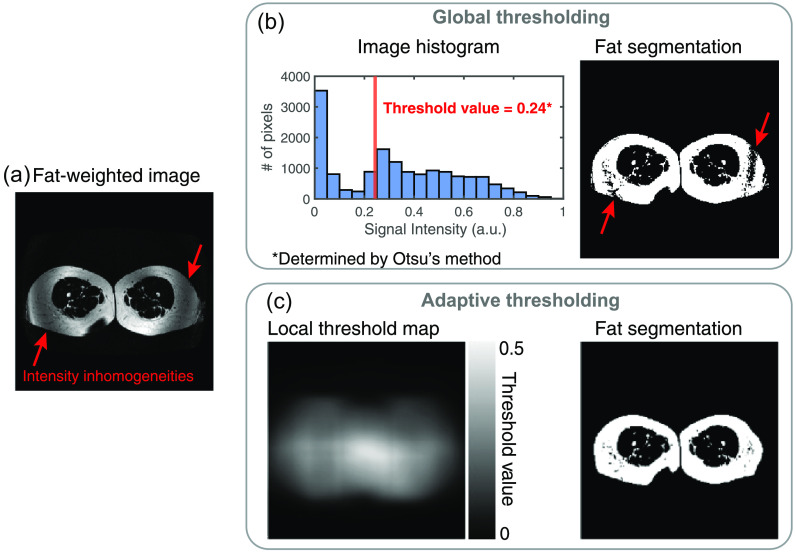
Global versus adaptive thresholding in extremity segmentation. (a) Signal intensity inhomogeneities (red arrows) can arise from poor $B_{0}$ shimming and signal drop off in regions farther from the scanner isocenter, which can be worsened in extremity scanning that has both a large in-plane and slice direction field of view. (b) Global thresholding techniques calculate an image histogram of signal intensities and determine a single threshold value that minimizes interclass variance between the foreground and background groups. However, the areas with signal drop off may be incorrectly classified as background pixels in the resulting segmentation (red arrows). (c) Adaptive thresholding can mitigate these false negative classifications by calculating a local threshold value for each pixel based on the mean intensity of a determined pixel neighborhood size. Applying the local threshold map produces a fat segmentation with improved foreground classification.

Fat-weighted and water-weighted anatomical contrasts enable classification of fat- and water-dominant voxels with intensity-based segmentation. Global intensity-based thresholding methods (such as Otsu’s method^18^) determine a single threshold value to segment an image histogram into background and foreground regions by minimizing interclass variance. However, signal intensity inhomogeneity (resulting from poor $B_{0}$ shimming and/or signal drop-off in regions of the field of view farther from the scanner isocenter) can confound global intensity-based methods in extremities (Fig 3). Thus, we applied a locally adaptive threshold method that determines a unique threshold value for each pixel in an image. Each pixel’s threshold value is determined based on the local mean intensity in a surrounding pixel neighborhood, or kernel. The kernel is moved around the image for pixel-wise calculations, and then the “map” of unique threshold values can be used to binarize the image, creating a foreground mask. Kernel matrix size must be chosen to avoid incorrect background classification with too small of a kernel and loss of fine details with too large of a kernel.^19^ To avoid these potential pitfalls, the square kernel size (s×s) was adjusted based on the approximate anterior-to-posterior size of the legs in each slice using

$$s=2\times \lfloor \frac{\left(H_{\mathrm{BOX}}\times \frac{3}{4}\right)}{2}\rfloor +1,$$ (1)

where $H_{\mathrm{BOX}}$ is the height of the 2D bounding box defined by the smallest rectangle that encloses the leg masks from the previous step (Fig. 2, step 2ab) and the ⌊ ⌋ brackets indicate the floor function. The kernel size equation was modified from a default equation in MATLAB that constrains the kernel size to a positive odd integer. The default equation is: $2\times \lfloor \frac{\text{size}(\text{Image})}{16}\rfloor +1$. However, this provided a kernel edge that was too small and resulted in incorrect classification of background pixels as foreground, and also did not adapt to the size of the legs (foreground) since it only considers total image size. Instead, by varying the scalar in the numerator of Eq. (1), the kernel size adapted based on the anterior–posterior distance of the legs in each slice ($H_{\mathrm{BOX}}$).

$H_{\mathrm{BOX}}$ is an approximate measure of anterior-to-posterior distance (see Fig. 2, step 1). Adaptive thresholding with the custom-fit kernel size was applied to each slice of water-weighted (Fig. 2, step 2a) and fat-weighted (Fig. 2, step 2b) images to produce a binary mask of all water components (water mask) and a binary mask of all fat components (fat mask), respectively.

#### 2.3.3 Muscle border segmentation

To segment skeletal muscle from the water mask, a series of Boolean operations, morphological operations, and flood-filling were applied (Fig. 2, step 3). The leg mask was eroded by a square kernel of 2×2  pixels and multiplied by the water mask to remove pixels in the thin outer skin layer. The fat mask was subtracted to remove fat-weighted components from the water mask, and spurious objects with areas <10  pixels were removed. Holes with area <18  pixels were filled to remove small holes in the mask while preserving the larger holes from the bones. As anatomy and morphology of the leg muscles change throughout the length of the lower extremities, morphological closing was carried out with two different pixel neighborhood sizes depending on location within the leg. A circular kernel with radius of 2 pixels was used for closing from the ankle to knee (approximate lower two stacks in lower extremity volume), and a larger radius of 4 pixels was used in the thigh (approximate upper two stacks). This resulted in a filled skeletal muscle mask with bones removed.

#### 2.3.4 SAT segmentation

SAT was defined as adipose tissue between the skin and muscle borders. To segment SAT from the total fat mask, the muscle mask (with holes filled from the muscle segmentation step 3 in Fig. 2) was subtracted from the fat mask to exclude most fat-weighted structures inside the muscle border (Fig. 2, step 4). In regions where the muscle does not surround the bone (and therefore does not get filled in during flood-filling of the muscle mask), this step is not sufficient for removing bone marrow from the fat mask. To account for this, morphological opening (erosion, followed by dilation) of the fat mask with a 2-pixel radius circular kernel was applied to remove connections between SAT and marrow, followed by exclusion of objects with circularity property >0.80, as the fatty bone marrow structures tended to be more circular in morphology than SAT. Finally, remaining objects of area <10  pixels were excluded, as these spurious pixels are not likely to be part of the SAT structure.

#### 2.3.5 IMAT and final segmentations

Finally, the intermuscular fat-weighted components (not previously defined as SAT), such as IMAT and cartilage, were subtracted from the muscle mask. This produced a final skeletal muscle mask for volume quantification (Fig. 2, step 5). The knee joint was segmented manually due to the morphological differences from the rest of the leg and large, irregular confounding areas of fat-weighted cartilage. All segmentations were visually checked and corrected as necessary.

Together, the functions in this algorithm that are directly modifiable are erosion and dilation kernel sizes, size-based exclusion regional areas (to remove spurious pixels), hole filling sizes, morphological closing neighborhood size, and object circularity. These parameters were determined by adjusting different values in a set of four training cases with slices representing the range of anatomy in the data set (i.e., the ankle and the thigh, two patients and two controls). The parameter value that identified the correct ROI in these training slices was used in the final algorithm for segmentation of the remaining independent test cases.

#### 2.3.6 Manual segmentations for algorithm validation

Manual ground-truth segmentations of a single slice in the calf and thigh of each participant were drawn by a single nonclinical imaging scientist with guidance from a board-certified radiologist (>10 years of experience in musculoskeletal radiology) using the MATLAB manual volume segmentation tool [volumeSegmenter()]. Manual segmentations were performed blinded to disease status.

### 2.4 Image and Statistical Analyses

The analysis objectives of this paper are to (1) quantify bilateral SAT and muscle volumes in multislice lower extremity CSE-MRI acquisitions, (2) evaluate performance of a semiautomatic segmentation routine compared with manual segmentations, and (3) assess differences in composition throughout the legs between participants with lipedema and BMI-matched controls. All statistical analyses were performed in R Statistical Software (version 4.1.0; R Foundation for Statistical Computing, Vienna, Austria). Descriptive statistics for continuous clinical parameters (age, BMI) were calculated and found to be normally distributed (Shapiro–Wilk test, P>0.05). To ensure the lipedema and control groups were matched for age and BMI, a two-sided unpaired Student’s t-test was used.

#### 2.4.1 Lower extremity composition quantification

For each slice, the following discrete metrics were calculated: SAT volume (mL), muscle volume (mL), and SAT-to-muscle volume ratio. Lower extremity adiposity in lipedema is approximately symmetric, therefore quantification of SAT and muscle composition was performed bilaterally, and the reported metrics represent the sum of both legs.^3^^,^^5^ Next, the stitched image volumes from thigh-to-ankles were split into decades: groups of slices each containing 10% of the total slices per subject, to normalize anatomical regions among participants of varying heights. The mean volume or ratio across the slices in each decade was reported to represent the mean per-slice metric in that corresponding anatomical region. The limb circumference of two regions (thigh and calf) was quantified on the left leg as the perimeter of the leg mask.

#### 2.4.2 Evaluation of segmentation routine

To evaluate the performance of the semiautomated segmentation algorithm throughout the leg, representative slices from uniform regions in the midthigh (decade 7) and superior calf (decade 3) were chosen for manual segmentation of the SAT and muscle ROIs. These were the same slices used for circumference quantification. On representative slices, Dice similarity coefficients (DSCs) were calculated for each ROI using

$$\mathrm{DSC}(A,G)=\frac{2|A\cap G|}{\left|A|+|G\right|},$$ (2)

where A is the algorithm’s segmentation result and G is the ground truth manual segmentation. The mean DSCs for lipedema and control subjects were normally distributed (Shapiro–Wilk test; P>0.05) and, thus, compared using a two-sided Student’s t-test (P≤0.05 required for significance) to assess whether the segmentation algorithm performed differently on images from participants with lipedema versus controls.

The final 3D whole-leg segmentations for each participant were reviewed blinded by a board-certified radiologist and given a pass or fail score and the number of slices in error recorded for each case.

#### 2.4.3 Comparisons between participants with lipedema and matched controls

The mean SAT volumes (mL), muscle volumes (mL), and SAT-to-muscle volume ratios of each decade were not normally distributed, and thus, compared using the nonparametric Mann–Whitney U test. Significance was defined as two-sided P≤0.05. Nonparametric effect size for nonnormally distributed data was calculated using the Wilcoxon test effect size (r) between 0 and 1.0.

## 3 Results

### 3.1 Participant Characteristics

Participants with lipedema (n=15) had a mean age=43.2±10 years, median age = 42.0 years, mean $\mathrm{BMI}=30.3\pm 4.3\;\mathrm{kg}/m^{2}$, and median $\mathrm{BMI}=29.4\;\mathrm{kg}/m^{2}$. Control participants without lipedema (n=13) had a mean age=42.2±13.5 years, median age = 41.0 years, mean $\mathrm{BMI}=28.8\pm 4.6\;\mathrm{kg}/m^{2}$, and median $\mathrm{BMI}=29.2\;\mathrm{kg}/m^{2}$. Participant groups were statistically matched for age (P=0.82) and BMI (P=0.39). All participants were female and self-reported White race.

### 3.2 Evaluation of Semiautomated Lower Extremity Segmentation Method

**Fig. 4:**
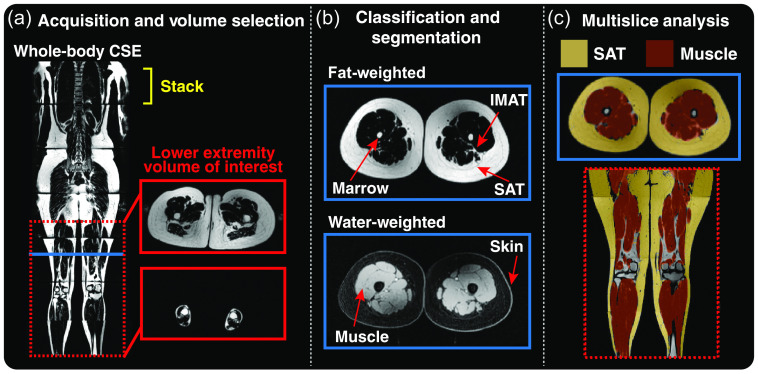
Lower extremity MRI semiautomated image analysis pipeline. (a) CSE-MRI was acquired in stacks from head-to-ankles to produce fat- and water-weighted images. Axial slices of the lower extremities were selected between the upper thigh and ankle (red lines). Duplicate slices resulting from overlap of neighboring stacks were removed. (b) Pixels were classified as fat or water from the respective fat- and water-weighted images. Regions including bone marrow, IMAT, and skin were removed while preserving SAT and skeletal muscle segmentations. (c) SAT (yellow overlay) and muscle (red overlay) masks are overlaid on an axial fat-weighted image (blue box), and a coronal representation of the lower extremities.

The semiautomated image segmentation pipeline for MRI from acquisition to analysis is summarized in Fig. 4. The total number of slices analyzed in each participant was 229±18 slices (mean ± standard deviation). Semiautomated image processing for each subject required ∼5  min, including selection of the standard upper and lower slices, slice stitching, and automated segmentation. The automated segmentation step had a mean runtime of 36.63±8.93  s (mean ± standard deviation).

DSC results comparing the semiautomated segmentation algorithm to manual segmentations are summarized in Table 1. For all 28 cases, in both the calf region (decade 3) and thigh region (decade 7), the mean DSCs for muscle and SAT segmentations were >0.95. In addition, the mean DSCs for participants with lipedema (n=15) and control participants (n=13) were not found to be significantly different (P>0.05) in either region. Whole-leg segmentations of all cases passed expert radiology review, and accurately identified SAT and muscle. When present, <2% of slices in each case had errors (errors affecting 1 to 2 slices per 3D volume), and on average <1% of slices analyzed had errors. When present, errors typically occurred at the ankle, where the SAT can be a thin layer, or in the upper thigh, where images were more prone to artifact. Further, one case had subcutaneous edema in the distal extremities, which caused misclassification of fat and water, due to the water-weighted edema, in two slices out of 208.

**Table 1:** Segmentation algorithm performance across leg. The DSC metrics are reported for the SAT region and skeletal muscle in both the thigh and the calf. The DSCs from control and lipedema groups were compared to test for statistical differences in the segmentation task performance. No significant differences in DSC for any region were found between healthy and diseased states.

		Group DSC (n=28)	Control DSC (n=13)	Lipedema DSC (n=15)	P-value*
SAT	Thigh	0.9754 ± 0.006	0.9732 ± 0.006	0.9772 ± 0.006	0.0849
	Calf	0.9598 ± 0.012	0.9575 ± 0.016	0.9617 ± 0.007	0.4111
Muscle	Thigh	0.9704 ± 0.009	0.9727 ± 0.006	0.9765 ± 0.005	0.1687
	Calf	0.9750 ± 0.004	0.9734 ± 0.005	0.9684 ± 0.010	0.0932

Note: DSC computed using Eq. (2). Values represent mean ± standard deviation.

* Student’s t-test with two-sided P≤0.05 required for significance.

### 3.3 Lower Extremity Adipose Composition in Patients with Lipedema

**Fig. 5:**
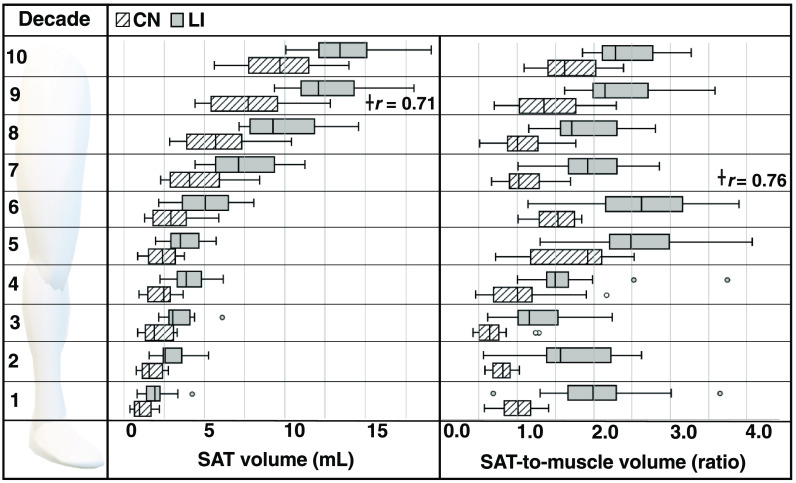
Decade-level comparison of imaging metrics. The mean SAT (left) and SAT-to-muscle volume ratio (right) for the control group (CN, hatch marks) and the lipedema group (LI, shaded) are visualized across 10 decades of the lower extremities. Both metrics were significantly higher (P≤0.01) in all decades. †Indicates decade with largest effect size for each metric (nonparametric Wilcoxon test effect size, r).

Decade-level imaging metrics throughout the legs are visualized in Fig. 5. Mean SAT volume and SAT-to-muscle volume ratio in participants with lipedema were significantly higher than matched controls in all decades throughout the leg (P≤0.05, Fig. 5). Muscle volume was not significantly different between the two groups for any decade. The greatest effect size of differential SAT volume was observed in the superior thigh region contained in decade 9 (r=0.710). The greatest effect size of SAT-to-muscle volume ratio was observed in the midthigh region contained in decade 7 (r=0.762).

A complete summary of limb circumferences and imaging metrics for the calf and midthigh regions are summarized in Table 2. Between circumference and imaging measurements, the difference in SAT-to-muscle volume ratio between patients with lipedema and controls demonstrated the largest effect size.

**Table 2:** Group results summary. Limb circumference and MRI-determined volume metrics for healthy controls and participants with lipedema are reported. Group comparisons for each measurement were made with nonparametric statistical tests and an effect size calculation to show the magnitude of differences. The metric of ratio of SAT-to-muscle volume had the highest effect size in both the thigh and the calf.

Measurement	Region	Control (n=13)	Lipedema (n=15)	P-value*	Effect size r
Limb circumference (cm)	Thigh	75.4 ± 11.3 (51, 91)	88.6 ± 8.1 (78, 102)	0.0020	0.593
	Calf	39.2 ± 4.34 (32, 46)	43.6 ± 3.60 (37, 49)	0.0012	0.457
SAT volume (mL)	Thigh	4.47 ± 1.88 (2.31, 8.43)	7.52 ± 2.30 (4.44, 11.26)	0.0022	0.562
	Calf	2.11 ± 0.86 (0.88, 3.34)	3.46 ± 1.02 (2.20, 6.10)	0.0018	0.570
Muscle volume (mL)	Thigh	4.02 ± 0.70 (2.63, 4.96)	3.85 ± 0.96 (2.22, 5.68)	0.4397	0.152
	Calf	2.99 ± 0.65 (2.10, 4.20)	2.83 ± 0.48 (2.23, 3.73)	0.6832	0.083
SAT to muscle ratio (unitless)	Thigh	1.09 ± 0.32 (0.67, 1.70)	2.00 ± 0.52 (1.02, 2.86)	<0.001	0.762
	Calf	0.72 ± 0.28 (0.42, 1.29)	1.26 ± 0.40 (0.62, 2.25)	<0.001	0.631

Note: Values represent mean ± standard deviation (minimum, maximum).

* Mann–Whitney U test with two-sided P≤0.05 required for significance.

**Fig. 6:**
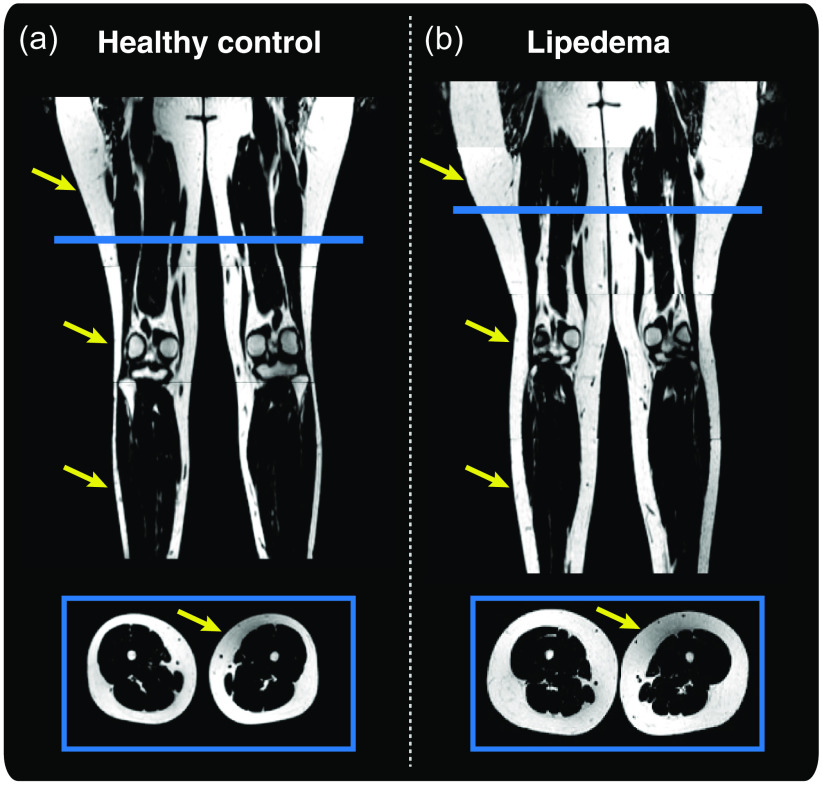
Representative example of (a) healthy female compared to a female patient with (b) lipedema. The healthy control is 28 years old with a BMI of $25.3\;\mathrm{kg}/m^{2}$ and the patient with lipedema is 23 years old with a BMI of $25.1\;\mathrm{kg}/m^{2}$. Structural measures of calf and thigh circumference are also similar (control versus lipedema values, calf circumference 37 cm versus 40 cm, thigh circumference 82 cm versus 86 cm). Lower extremity CSE-MRI reveals apparent thickened SAT in lipedema extending from thighs to ankles (yellow arrows). This is also observable on the transverse slice at the thigh level (blue box). SAT volume can be quantified by image analysis of musculoskeletal composition. In the midthigh region (decade 7): the mean SAT volume is greater for lipedema versus control 5.90 versus 3.89 mL, whereas mean muscle volume for lipedema versus control is 3.45 versus 4.16 mL. The SAT/muscle volume ratio in the midthigh is also greater in lipedema versus control 1.73 versus 0.94. Note: All stacks were rendered with an identical grayscale window/level, and image proportions were preserved.

A representative example of lower extremity CSE-MRI images is presented in Fig. 6 from a participant with lipedema alongside a healthy female without lipedema but with similar age, BMI, calf circumference, and thigh circumference. Elevated SAT quantity is observable along the length of the legs in the patient with lipedema in the coronal view and a transverse view in the thighs.

## 4 Discussion

In this study, whole-body CSE-MRI acquisitions were acquired, and semiautomated image analysis procedures were developed to test the hypothesis that SAT volume is elevated throughout the lower extremities of patients with lipedema compared to females without lipedema but with similar age and BMI. We designed a semiautomated workflow for image volume selection and segmentation of SAT and skeletal muscle from lower extremity CSE-MRI acquisitions, and analysis consisting of SAT volume and SAT-to-muscle volume ratio quantification. The semiautomated workflow provided segmentations of lower extremity SAT and muscle that had a high degree of spatial overlap with manual segmentation (mean DSC>0.95) in the calf and thigh. Elevated SAT volume (P<0.01) and SAT-to-muscle volume ratio (P<0.001) were observed in all sections of the lower extremities. The greatest effect size for differentiating SAT deposition in lipedema compared to female controls was demonstrated in the thighs using the SAT-to-muscle volume ratio (effect size = 0.762). Herein, we demonstrate an extension to our previously accepted work of SPIE Medical Imaging.^20^

### 4.1 Evaluation of Semiautomated Segmentation Method

Lower extremity MRI acquisitions may be useful in revealing differential internal metrics in the lower extremities of individuals with lipedema. Given the challenges of working with multislice data and the varying anatomical morphology throughout the leg, development of automated techniques greatly improved efficiency for quantifying the musculoskeletal composition. The methods described require <5  min to perform manual volume selection and automated segmentation, including a mean runtime of 36.63±8.93s for the automated algorithm. The segmentation algorithm is built in a MATLAB environment and is comprised entirely of classical image processing techniques (thresholding, active contouring, flood-filling, morphological operations, and Boolean operations) available in the MATLAB image processing toolbox.

The algorithm was found to be comparable to manual segmentation in two key regions in the leg used for body composition analysis (calf and thigh) with mean DSCs for both SAT and muscle >0.95. The algorithm performed similarly in both participants with lipedema and controls in the calf and thigh, yielding DSCs not significantly different between the two groups for either region of interest. The DSC calculation was performed between a filled muscle mask with only bones removed, compared to the final segmented mask that excluded fat-weighted intermuscular components (adipose tissue, fascia, and cartilage). This was because manual segmentations of muscle did not remove these intermuscular components due to the unreliability of nonexpert manual segmentations to classify these small structures. Though this is a limit of the validation, it highlights a strength of the segmentation algorithm, which is capable of segmenting muscle while excluding these nonmuscular components, enhancing the accuracy of the automated algorithm.

There is a need for a more automated methodology to segment the varying anatomy of lower extremity MRI data for the purposes of internal composition metrics to study the unique lower extremity pathology of lipedema. Existing semiautomated methods for segmenting musculoskeletal tissues of whole-body data include atlas-based, thresholding, clustering, active contours, and morphological segmentation techniques, deep learning, as well as combinations of these methods. Fully automated techniques to segment the lower extremities exist; however, current applications are suitable for a single slice in the thigh or calf, sometimes with only one leg in the field of view.^21^^–^^23^ Thus, our method addresses the need for automated multislice segmentations of images contained in rapid lower extremity acquisitions from Dixon MRI. Advantages of this method include ability to synthesize the large amount of data contained in whole-leg imaging, reduce the time-consuming task of manual segmentation, and avoid interrater variability.^16^^,^^21^ Fully-automated deep learning and atlas-based segmentation techniques may be impractical for applications in lipedema as a result of the uncommon diagnosis of lipedema that limits sample size, and the high anatomical variability in lower extremity soft tissue and vascular characteristics of this disease. It is possible that our classical approach could provide initial segmentations for a deep learning approach, similar to work by Yang et al.^24^ for single slice thigh computed tomography images.

### 4.2 Lipedema has Distinct SAT Accumulation throughout the Lower Extremities

Significant clinical questions regarding lipedema-related SAT accumulation compared to obesity have limited the diagnostic and treatment options for this debilitating disease. The clinically relevant result arising from this work is that SAT volume and SAT-to-muscle volume ratio are elevated throughout the lower extremities in women with lipedema compared to healthy women matched for BMI, whereas skeletal muscle volumes are not significantly different. In comparison to calf or thigh circumferences, the nonparametric effect sizes are larger for the MRI-derived internal metrics (SAT volume, SAT-to-muscle ratio), indicating these metrics are more powerful at differentiating the unique lower extremity composition in lipedema. Lower extremity CSE-MRI was acquired using conventional sequences at clinical field strength within a 10-min acquisition and should be available to perform at most imaging medical centers. Our semiautomated image processing pipeline provided objective quantification of SAT distribution relevant to distinguishing lipedema from obesity.

Findings in this study corroborate previous observations from more localized Na23/H1-MRI of SAT quantity in the calf that also revealed elevated tissue sodium content in this region of patients with lipedema.^14^^,^^15^ The midsuperior calf region, contained in decades 3 and 4 of this study’s imaging procedures, is a consistent region of differential MRI-based body composition in lipedema. In this study, we additionally learned that the thigh region demonstrates the greatest effect sizes of differential SAT volume and SAT-to-muscle volume ratio between lipedema and controls. This represents a promising ROI to inform the structure and distribution of lipedema SAT pathology and may inform future studies investigating lipedema-related and nonlipedema related adipose tissue. Additional structural and molecular imaging techniques (e.g., high spatial-resolution $T_{1}$- and $T_{2}$-weighted MRI, sodium Na23-MRI, H1 relaxometry) and statistical image analysis approaches (e.g., texture quantification) are being investigated to develop discriminative, noninvasive MRI biomarkers of lipedema. Recently, vascular imaging revealed unique spatial patterns of fluid retention in lipedema SAT and could be incorporated into a multimodal imaging approach to disambiguate potential vascular etiology of SAT deposition.^25^ The relationship of such radiological biomarkers to clinical symptomatology and functional impairments is also of interest to better understand the complex disease mechanisms of lipedema.

### 4.3 Limitations

The adaptive kernel size chosen to perform local thresholding enables robustness to changing limb size and intensity inhomogeneity; however, the selection of this parameter in Eq. (1) can affect method generalizability to image resolutions different than utilized in this standard acquisition protocol. Thus, for different acquisition protocols, this equation may require modification to the scalar that is multiplied by the bounding box height term. The CSE-MRI method utilized is a two-point technique with two echo times and was chosen for acquisition speed and inherent separation of signal from fat- and water-weighted species. CSE-MRI sequences with longer echo trains could provide $B_{0}$ maps, enabling main field inhomogeneity correction to improve image quality for segmentation. Improved quantitation of fat-fraction maps would also be feasible with multiecho acquisitions and enable segmentation by soft clustering with a higher sensitivity to boundary voxels containing both fat and water signal. However, the methods described herein are sufficient to elucidate significant differences with high statistical power (r>0.60) between the SAT-to-muscle volume ratio of participants with lipedema versus the matched controls throughout the leg. Metrics of SAT volume and SAT-to-muscle volume ratio merit further validation in a larger cohort. For analysis along the lower extremities, slices for each participant were partitioned into decades, each containing 10% of the slices. Although this allows for an efficient means of obtaining approximate anatomical comparisons of metrics between participants of different heights, it assumes the proportions of the legs are the same for all participants.

Though this report of lower extremity CSE-MRI and multislice image analysis was performed in 28 participants, it represents a well-characterized study in this uncommonly diagnosed disease. Even though there are 28 subjects represented, there are on average 229 slices analyzed in each person. As the legs are primarily affected by fat deposition in lipedema, it was important to analyze a larger set of anatomy. In each of these slices and across the extremity, we devised a strategy to analyze the whole-leg adipose deposition in an automated, objective manner. This image analysis strategy enables us to begin to address a clinically meaningful question: where in the legs does adipose deposition vary the most in participants with lipedema compared with control subjects? This novel method advances our understanding for prior studies could not address this question in a single slice analysis. This method is being used to compare a specific disease to a well-matched control cohort and would require validation in a broader range of anatomies and patient populations to apply it to other applications.

## 5 Conclusions

Lipedema is an underdiagnosed patient population characterized by disproportionate SAT deposition in the legs. This work applied lower extremity CSE-MRI in a whole-leg manner for body composition analysis relevant to lipedema. We implemented a multislice semiautomated image segmentation and quantitative analysis pipeline. This algorithm was applied in a pilot study to evaluate cases with lipedema and age- and BMI-matched controls. Findings reveal uniquely elevated SAT volume and SAT-to-muscle volume ratio throughout the lower extremities of patients with lipedema compared with controls. Results highlight lower extremity SAT and muscle composition analysis as a promising tool for the differential diagnosis and study of lipedema. Future work will seek to enlarge the sample size for confirmation and relate internal composition findings by MRI to symptomatology and functional impairments in lipedema. This study contributes to our long-term goal of identifying and implementing objective tools for the diagnosis of lipedema.

## Code, Data, and Materials Availability

Code can be made available upon request by contacting the corresponding author (shannon.l.taylor@vanderbilt.edu).
